# ASRpro: A machine‐learning computational model for identifying proteins associated with multiple abiotic stress in plants

**DOI:** 10.1002/tpg2.20259

**Published:** 2022-09-13

**Authors:** Prabina Kumar Meher, Tanmaya Kumar Sahu, Ajit Gupta, Anuj Kumar, Sachin Rustgi

**Affiliations:** ^1^ ICAR‐Indian Agricultural Statistics Research Institute New Delhi India; ^2^ ICAR‐National Bureau of Plant Genetic Resources New Delhi India; ^3^ Dep. of Microbiology and Immunology Dalhousie Univ. Halifax Nova Scotia Canada; ^4^ Laboratory of Immunity Shantou Univ. Medical College Shantou PRC; ^5^ Dep. of Plant and Environmental Sciences, Pee Dee Research and Education Centre Clemson Univ. Florence SC USA

## Abstract

One of the thrust areas of research in plant breeding is to develop crop cultivars with enhanced tolerance to abiotic stresses. Thus, identifying abiotic stress‐responsive genes (SRGs) and proteins is important for plant breeding research. However, identifying such genes via established genetic approaches is laborious and resource intensive. Although transcriptome profiling has remained a reliable method of SRG identification, it is species specific. Additionally, identifying multistress responsive genes using gene expression studies is cumbersome. Thus, endorsing the need to develop a computational method for identifying the genes associated with different abiotic stresses. In this work, we aimed to develop a computational model for identifying genes responsive to six abiotic stresses: cold, drought, heat, light, oxidative, and salt. The predictions were performed using support vector machine (SVM), random forest, adaptive boosting (ADB), and extreme gradient boosting (XGB), where the autocross covariance (ACC) and *K*‐mer compositional features were used as input. With ACC, *K*‐mer, and ACC + *K*‐mer compositional features, the overall accuracy of ∼60–77, ∼75–86, and ∼61–78% were respectively obtained using the SVM algorithm with fivefold cross‐validation. The SVM also achieved higher accuracy than the other three algorithms. The proposed model was also assessed with an independent dataset and obtained an accuracy consistent with cross‐validation. The proposed model is the first of its kind and is expected to serve the requirement of experimental biologists; however, the prediction accuracy was modest. Given its importance for the research community, the online prediction application, ASRpro, is made freely available (https://iasri‐sg.icar.gov.in/asrpro/) for predicting abiotic SRGs and proteins.

## Core Ideas

- Identification of multiple abiotic SRGs is vital to breeding for stress tolerance.
- We developed a computational model using ML algorithms to predict abiotic stress‐responsive proteins.
- Being the first of its kind tool, expected to supplement the wet experiments for identifying SRGs.
- The online prediction tool ASRpro (https://iasri‐sg.icar.gov.in/asrpro/) is made freely available to the community.

**Abbreviations**
ACC: autocross covariance
ADB: adaptive boosting
auPRC: area under the precision‐recall curve
auROC: area under the receiver operating characteristic curve
GWAS: genome‐wide association study
ML: machine learning
RFE: recursive feature elimination
SRG: stress‐responsive gene
SVM: support vector machine
XGB: extreme gradient boosting

## 1 INTRODUCTION

Any deviation from the optimum growing conditions preventing plants from attaining the full genetic potential for reproduction and growth constitutes stress (Boscaiu et al., 2008). As far as abiotic stresses are concerned, they are caused by physical or chemical stressors in the crops’ immediate environment. The crop growing environment is unpredictable because of constant climatic changes making abiotic stress a serious threat to plant growth and development (Cao et al., 2015). Abiotic stresses adversely affect crop production (Atkinson & Urwin, 2012), reducing yield by 50% (Qin et al., 2011). Besides crop yield losses, it also poses a threat to plant biodiversity. Cold, drought, heat, light, oxidative, and salt stresses are commonly encountered abiotic stresses that unfavorably affect plant health (Paul et al., 2014). Understanding the plant response to such abiotic stresses at the molecular level would help develop crop cultivars with improved stress tolerance. In particular, discovering stress‐responsive genes (SRGs) and proteins is crucial to selecting stress tolerance in plants (Ma et al., 2012).

With the availability of whole‐genome sequences of several plant species, the discovery of SRGs at a genome‐wide level has become feasible. For instance, Famoso et al. (2011) performed a genome‐wide association study (GWAS) using 383 rice (*Oryza sativa* L.) accessions and identified 48 aluminum‐stress responsive genomic regions. Further, the next‐generation sequencing technologies have expanded the scope to discover SRGs. Specifically, transcriptome profiling was adopted on many occasions for SRG identification (Tillett et al., 2011; Atkinson et al., 2013; Zhang et al., 2017; Shu et al., 2018; Sui et al., 2018; Yang et al., 2018; Zhou et al., 2018; Smita et al., 2020). The SRGs were also identified in many plant species through evolutionary analysis of the gene families (Mochida et al., 2009, 2010).

As far as computational identification of abiotic SRGs is concerned, Paul et al. (2014) proposed an approach for classifying up‐ and downregulated genes in response to heat, cold, drought, and salinity stresses. However, no computational tool is available to identify genes and proteins responsive to different abiotic stresses in plants. Although transcriptome profiling is reliable in predicting abiotic SRGs, it is species specific and resource intensive. Identification of abiotic stress‐associated markers through GWAS was performed, and the information was used to predict candidate genes contributing to abiotic stress tolerance. However, GWAS provides less accuracy with low heritability traits and when the markers are not in linkage disequilibrium with the quantitative trait loci. Given these factors, it is desirable to develop a computational method for predicting abiotic SRGs. Based on the machine‐learning (ML) algorithm, a computational method has been developed in this study to predict multiple abiotic SRGs using high throughput sequence data. The proposed methodology is believed to supplement transcriptome profiling, comparative genomics, and GWAS for abiotic SRG recognition. The order of the abiotic stresses (cold, drought, heat, light, oxidative, and salt) will remain the same hereafter.

**Core Ideas**

- Identification of multiple abiotic SRGs is vital to breeding for stress tolerance.
- We developed a computational model using ML algorithms to predict abiotic stress‐responsive proteins.
- Being the first of its kind tool, expected to supplement the wet experiments for identifying SRGs.
- The online prediction tool ASRpro (https://iasri‐sg.icar.gov.in/asrpro/) is made freely available to the community.

## 2 MATERIALS AND METHODS

### 2.1 Sequence data

Stress‐responsive proteins for six abiotic stresses—cold, drought, heat, light, oxidative, and salt—were retrieved from four publicly available databases: ASRGDB (Borkotoky et al., 2013), PSPDB (Anil Kumar et al., 2014), STIFDB2 (Naika et al., 2013), and DroughtDB (Alter et al., 2015). Sequence distribution over different databases for each stress is given in Table 1. A total of 1,089; 1,250; 342; 275; 437; and 1,184 protein sequences were retrieved for the respective abiotic stresses.

**TABLE 1:** Summary of the sequences retrieved from the public databases, ASRGDB, PSPDB, STIFDB2, and DroughtDB

Database	Cold	Drought	Heat	Light	Oxidative	Salt
ASRGDB	15	31	28	11	130	138
DroughtDB	–	188	–	–	–	–
PSPDB	–	215	249	264	267	14
STIFDB2	1,074	816	65	–	40	1,032
No. total sequence	1,089	1,250	342	275	437	1,184

### 2.2 Positive and negative datasets

Sequences in more than one stress treatment were excluded, and the sequences that appeared exclusively in a single stress treatment were retained for the analysis. To remove the bias attributable to homologous sequences in each category, the sequences with >40% identical bases were excluded using CD‐HIT (cluster database at high identity) (Fu et al., 2012). For a given stress category, sequences appearing exclusively in that category constituted the positive dataset, and the sequences appearing under the remaining five stresses together built the negative dataset. The negative datasets were also subjected to the removal of redundancy because of homologous sequences followed by the exclusion of sequences with nonstandard residues, such as B, J, O, U, X, and Z. The numbers of sequences for the positive sets were 444, 394, 110, 122, 127, and 348, and for the negative set, 1,339; 1,397; 1,595; 1,576; 1,577; and 1,417, respectively, for the abiotic stresses. A summary of the positive and negative datasets is given in Table 2.

**TABLE 2:** Summary of the positive and negative datasets

Category	Cold	Drought	Heat	Light	Oxidative	Salt
Single stress^a^	558	543	167	204	265	526
Positive set^b^	444	394	110	122	127	348
Negative‐I^b^	1,339	1,397	1,595	1,576	1,577	1,417

^a^ Number of sequences belonging to single stress only.

^b^ Number of sequences with <40% sequence identity.

### 2.3 Feature generation

Feature generation is an essential component of the computational prediction using biological sequence data as far as achieving higher prediction and classification accuracy is concerned. This study used *K*‐mer compositional features and autocross covariance (ACC) features to represent the protein sequences into numeric feature vectors.

#### 2.3.1 *K*‐mer compositional features

To a large extent, a protein's amino acid composition determines its physicochemical properties and function. However, the amino acid composition considers the information for the whole protein sequence and ignores the sequence order (Yao et al., 2019). Therefore, *K*‐peptide compositional features such as di‐ and tripeptide compositions have also been used for predictions, and better results were obtained than amino acid composition. For *K*‐mer compositional features (*K*‐mer), the dimension of the numeric feature vector will be 20^K^. In this study, we considered *K* = 1, *K* = 2, and *K* = 3; hence, the total number of features generated was 20^1^ + 20^2^ + 20^3^ = 8,420, a high‐dimensional feature vector.

#### 2.3.2 Autocross covariance features

Autocross covariance of the physicochemical properties, that is, hydrophobicity, hydrophilicity, and mass, was computed and used to encode the variable‐length protein sequences into fixed‐length numerical vectors. Since its inception, the ACC features have been employed by many investigators in bioinformatics (Guo et al., 2006; Zeng et al., 2009; Zhao et al., 2012; Li et al., 2015; Liu et al., 2016; Qiu et al., 2017). The ACC features are a combination of two features: autocovariance features (ACF) and cross‐covariance features (CCF). The autocovariance features and cross‐covariance features measure the covariance between the same and different physicochemical properties, respectively, along the protein sequences (Dong et al., 2009). The ACC features capture the average interaction between amino acids separated by certain residues (called Lag) in the sequence, making it possible to identify patterns by taking neighboring effects into account. The precise computational procedure for autocovariance features and cross‐covariance features are as follows:

For a given sequence Z of n amino acid residues long, the autocovariance (AC) with Lag value lg can be computed as $\mathrm{AC}\left(j,\mathrm{lg}\right)=\frac{1}{n-\mathrm{lg}}\sum_{i=1}^{n-\mathrm{lg}}\left(Z_{ij}-\frac{1}{n}\sum_{i=1}^{n}Z_{ij}\right)\left[Z_{(i+\mathrm{lg})j}-\frac{1}{n}\sum_{i=1}^{n}Z_{ij}\right]$, where *Z_ij_* is the value of the *j*th descriptor at *i*th position (*i* = 1, 2, 3, …, *n*; *j* = 1, 2, 3, …, *P*). Using the same notation, the cross‐variance (CC) can be calculated as $\mathrm{CC}\left(j,k,\mathrm{lg}\right)=\frac{1}{n-\mathrm{lg}}\sum_{i=1}^{n-\mathrm{lg}}\left(Z_{ij}-\frac{1}{n}\sum_{i=1}^{n}Z_{ij}\right)\left[Z_{(i+\mathrm{lg})k}-\frac{1}{n}\sum_{i=1}^{n}Z_{ik}\right]$, where *i* denotes the position in the sequence and *Z_ij_* and *Z_ik_* are the values of the *j*th and *k*th descriptors, respectively, at *i*th position (*i* = 1, 2, 3, … *n*; *j, k* = 1, 2, 3, …, *P*; *j* ≠ *k*). We considered the default lg = 2 and the total number of ACC features generated were 1,641.

### 2.4 Feature selection

The high‐dimensional feature set may produce noisy and redundant data resulting in less classification accuracy. Additionally, prediction with a high‐dimensional feature set may take longer. Thus, we adopted the support vector machine (SVM) recursive feature elimination (RFE) method (Guyon et al., 2002) to reduce the dimension and eliminate the redundant and noisy features. Doing so makes the computational procedure less complex and improves the prediction accuracy (Aksu et al., 2010; Huang et al., 2014). In SVM‐RFE, the features were eliminated in an iterative process where the square of the SVM classifier weights were used as a measure for the same. The most important features were removed in the last iteration, whereas the least important one was removed in the first iteration. Here, we selected the number of features with which the highest classification accuracy was obtained, similar to the existing studies (Guyon et al., 2002; Lin et al., 2012). The ‘sigFeature’ R‐package (Das et al., 2020) was employed to implement the SVM‐RFE method.

### 2.5 Prediction using machine‐learning methods

Four different ML algorithms, such as SVM (Cortes et al., 1995), random forest (Breiman, 2001), extreme gradient boosting (XGB) (Chen & Guestrin, 2016), and adaptive boosting (ADB) (Freund & Schapire, 1999), were employed for the prediction. Since these algorithms have been well described in the literature, the working principle of these algorithms was not elaborated further. As far as implementation of these algorithms is concerned, the R‐packages e1071 (Meyer et al., 2019), randomForest (Liaw, 2002), xgboost (Chen et al., 2015), and adabag (Alfaro et al., 2013) were used for implementing the SVM, random forest, XGB, and ADB ML methods, respectively. The algorithms were first implemented with default parameter settings, and the final predictions were made using the selected algorithm with which higher accuracy was obtained.

### 2.6 Cross‐validation and assessment metrics

**FIGURE 1:**
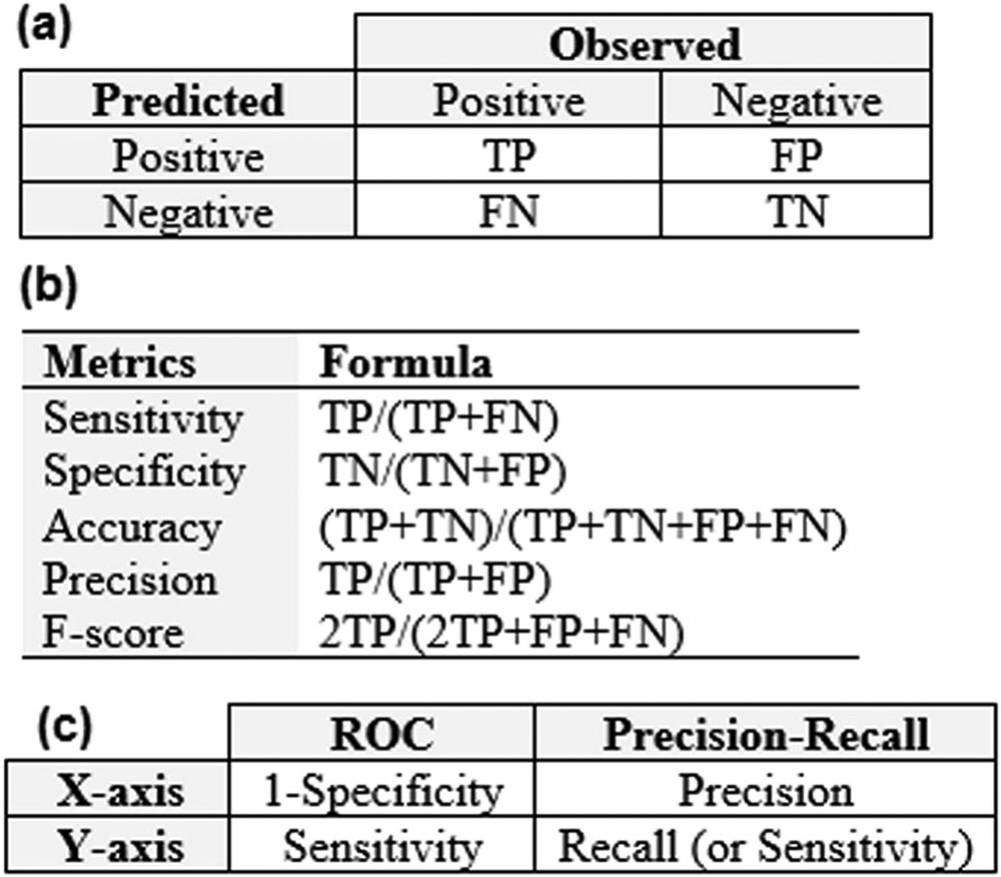
Representation of (a) confusion matrix, (b) different performance metrics, and (c) receiver operator characteristic (ROC) and precision‐recall measures. All the performance metrics, including ROC and precision‐recall curves, are the function of the confusion matrix. TP, true positive; TN, true negative; FP, false positive; FN false negative

**FIGURE 2:**
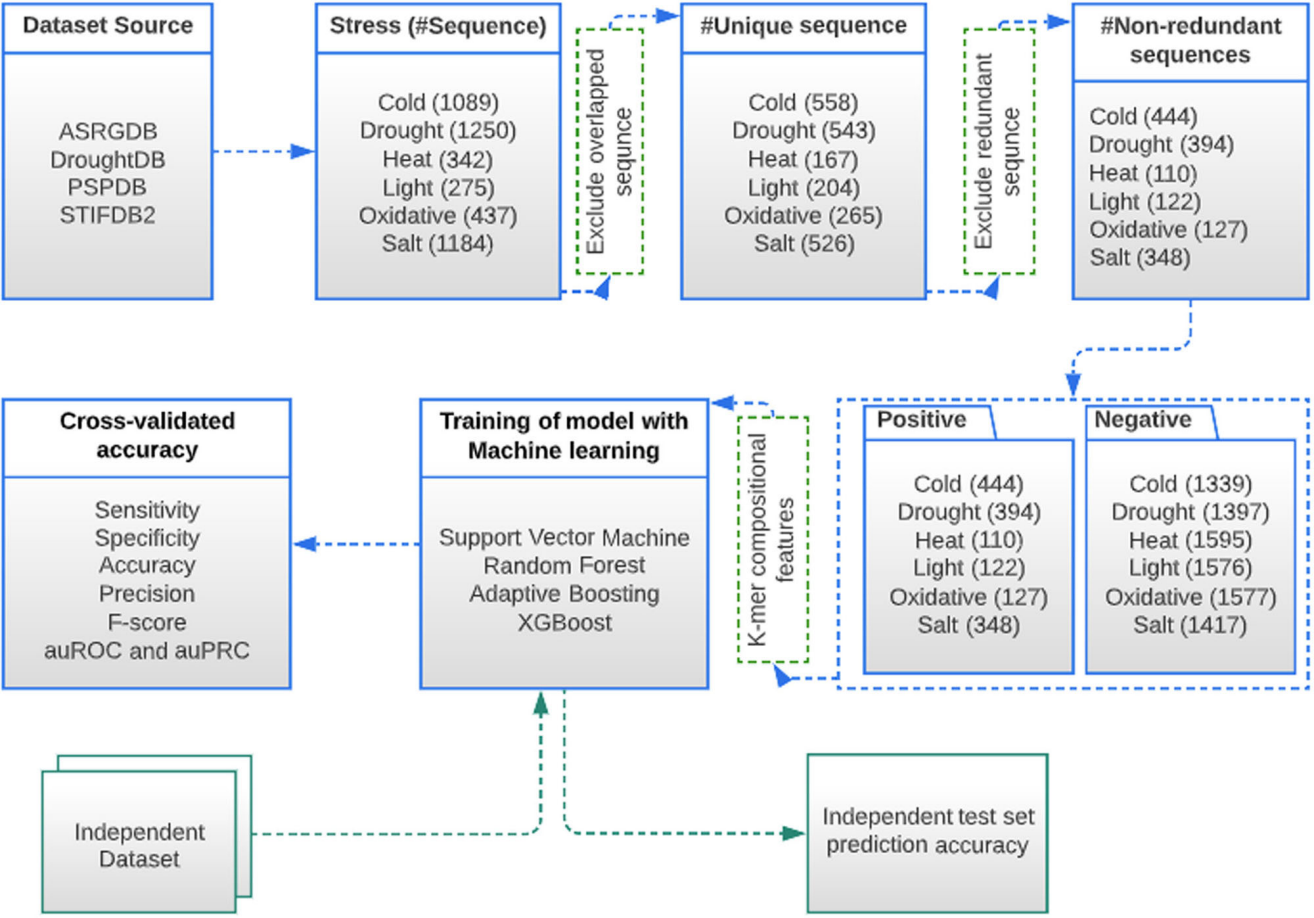
A flow diagram depicting the step in the prediction pipeline developed to identify stress‐responsive proteins

Using one training and one test set may result in a higher bias in the prediction accuracy. Therefore, the process of training and testing should be done multiple times and that too with the changes in the distribution of the training and test datasets. Here, we employed a fivefold cross‐validation technique to validate the effectiveness of the prediction model. In this procedure, the entire dataset was split into five equal‐sized sets, where the model was trained with four sets, and its efficiency was tested with the remaining one set. This process was repeated until each of the five sets was served as the test set. The performance metrics were computed by taking the average over all the five folds. All these performance metrics are the function of the confusion matrix (Figure 1) that comprises the information of true positive, true negative, false positive, and false negative. The metrics, such as sensitivity, specificity, accuracy, precision, and *F* score (Figure 1), were employed to measure the accuracy. Additionally, the area under the receiver operating characteristic curve (auROC) (Krzanowski & Hand, 2009) and area under the precision‐recall curve (auPRC) (Davis & Goadrich, 2006) were also computed to measure the performance of the predictors. The receiver operating characteristic curve can be obtained by plotting the false positive rate (1‐specificity) on the *x* axis and true positive rate (sensitivity) on the *y* axis, whereas the precision‐recall curve can be plotted by taking precision and recall in the *x* and *y* axes, respectively (Figure 1). A flow diagram comprising all the steps of the proposed approach is shown in Figure 2.

## 3 RESULTS

### 3.1 Preliminary analysis of the sequence data

**FIGURE 3:**
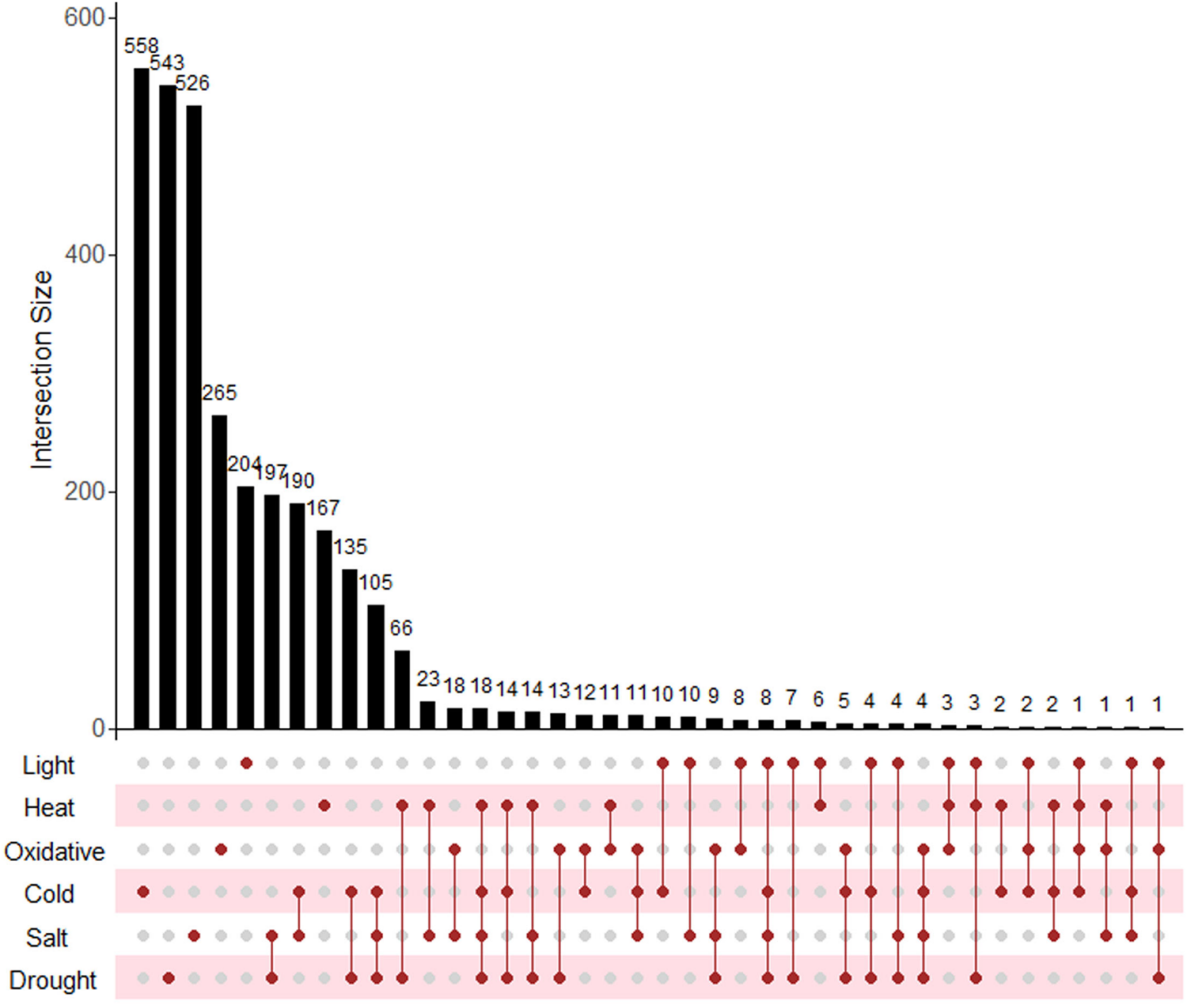
Intersection plot showing the number of overlapped sequences in different stresses. The same protein sequences can be found present in two, three, and four stresses

The analysis of the positive sequence dataset suggested that most of the sequences were associated with more than one stress (Figure 3). In other words, the same sequences were found to be present in two, three, four, and even more than four stresses. In particular, many sequences were found to be common among cold, drought, salt, and heat stresses. For instance, 197, 190, and 135 sequences were common between drought and salt, cold and salt, and cold and drought, respectively. Similarly, 105 sequences were common among cold, drought, and salt stresses. Additionally, 18 sequences were common among cold, drought, salt, and heat stresses (Figure 3).

### 3.2 Feature selection analysis

**FIGURE 4:**
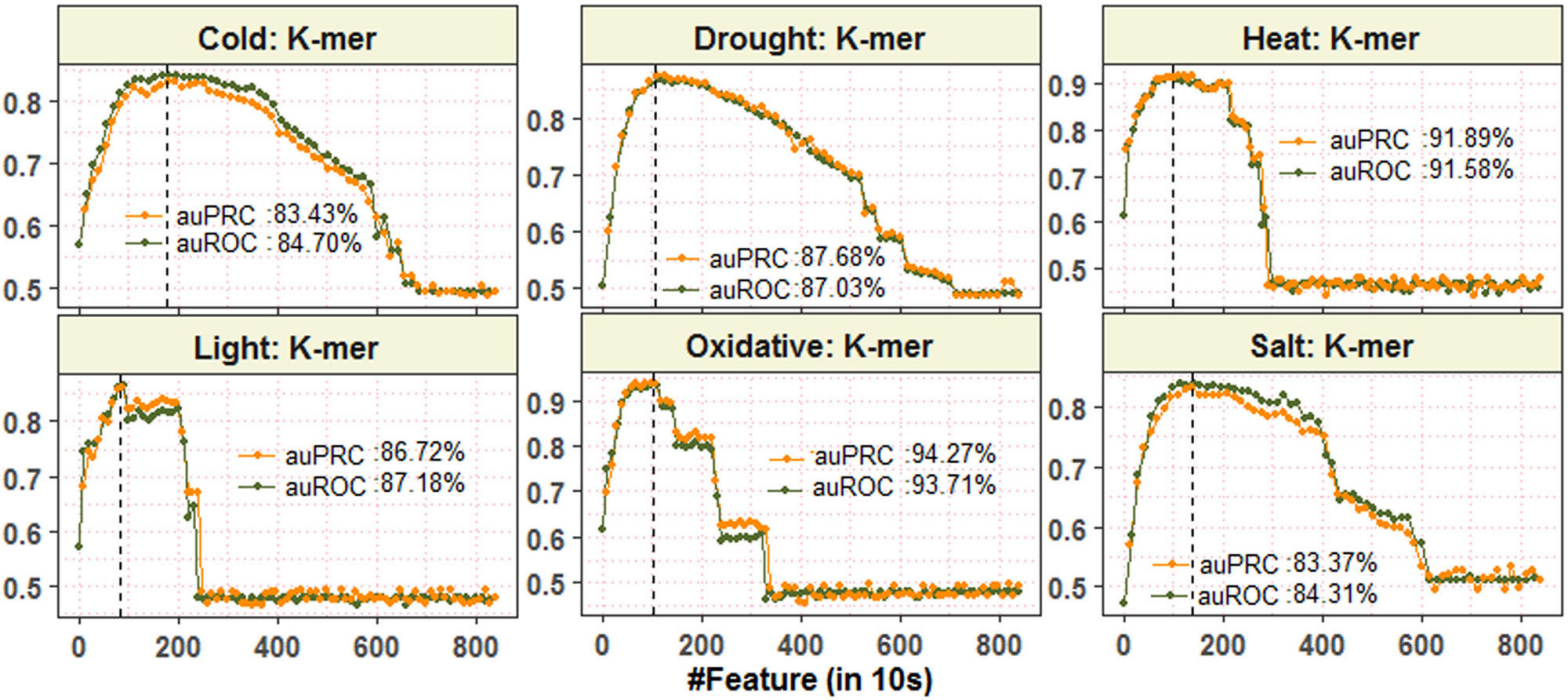
Plots of area under receiver operating characteristics curve (auROC) and area under precision‐recall curve using the ranked *K*‐mer compositional features obtained with the support vector machine (SVM)‐recursive feature elimination (RFE) feature selection approach

The feature selection was performed on 50% randomly drawn sample observations from the positive and negative sets. Three feature sets—*K*‐mer, ACC, and ACC + *K*‐mer—were considered. Optimum feature sets were different for different stress categories, where the optimum feature set comprised the selected number of features with which the highest auROC and auPRC were obtained. Out of 8,420 *K*‐mer compositional features, the selected optimum features were 1,790; 1,050; 990; 850; 1,040; and 1,400, respectively, for cold, drought, heat, light, oxidative, and salt stresses (Figure 4). The optimum feature sets containing 40, 50, 70, 50, 40, and 40 selected features were obtained for different stress categories out of 1,641 ACC features (Supplemental Figure S1). For the combined features (8,420 + 1,641), the features selected for the optimum feature sets were 3,280; 2,510; 80; 70; 40; and 3,200 for different stress categories (Supplemental Figure S1). It was also observed that the accuracy was closer to the random guess (∼50%) when all the features were used for prediction (Figure 4).

### 3.3 Prediction analysis using SVM

To avoid the biased prediction accuracy toward the negative set having a larger number of observations vs. the positive set, the prediction was performed by using a balanced dataset prepared by taking all the instances of the positive set and randomly drawn the same number of instances from the respective negative set. The predictions for different stress categories were performed with the respective selected feature sets, and the prediction accuracy was measured following a fivefold cross‐validation approach. Overall accuracy for the respective stress categories was found to be higher with the selected *K*‐mer compositional features (79.91, 77.31, 86.36, 77.50, 86.00, and 75.94%) than ACC (59.32, 65.64, 77.73, 72.50, 74.00, and 60.87%), and *K*‐mer + ACC (63.18, 61.79, 78.64, 70.42, 69.20, and 62.17%) feature sets (Table 3). For *K*‐mer, ACC, and *K*‐mer + ACC feature sets, the sensitivities for cold, drought, and oxidative stresses were found to be higher than their respective specificities. On the other hand, the specificities of heat, light, and salt were found to be higher than their respective sensitivities with a few exceptions (Table 3). Further, the overall prediction accuracy for the heat (*K*‐mer, 86.36%; ACC, 77.73%; and *K*‐mer + ACC, 78.64%), light (*K*‐mer, 77.50%; ACC, 72.50%; and *K*‐mer + ACC, 71.68%), and oxidative (*K*‐mer, 86.00%; ACC, 74.00%; and *K*‐mer + ACC, 69.20%) stresses were found to be higher than that of cold (*K*‐mer, 75.91%; ACC, 59.32%; and *K*‐mer + ACC, 63.18%), drought (*K*‐mer, 77.31%; ACC, 65.64%; and *K*‐mer + ACC, 61.49%), and salt (*K*‐mer, 75.94%; ACC, 60.87%; and *K*‐mer + ACC, 62.17%) stresses (Table 3).

**TABLE 3:** Prediction accuracy of support vector machine (SVM) with the optimum feature set. Prediction accuracy was computed following a fivefold cross‐validation approach, where the SVM‐recursive feature elimination‐based selected features were utilized for prediction

Stress	Sen	Spe	Acc	Pre	*F* score	auROC	auPRC
***K*‐mer**							
Cold	78.64	73.18	75.91	74.57	76.55	84.61	82.95
Drought	81.28	73.33	77.31	75.30	78.18	87.06	87.91
Heat	80.91	91.82	86.36	90.82	85.58	90.88	91.30
Light	65.83	89.17	77.50	85.87	74.53	85.95	85.82
Oxidative	90.40	81.60	86.00	83.09	86.59	93.62	94.00
Salt	72.75	79.13	75.94	77.71	75.15	84.21	83.11
**ACC**							
Cold	60.23	58.41	59.32	59.15	59.68	63.11	60.18
Drought	66.15	65.13	65.64	65.48	65.82	68.74	68.20
Heat	79.09	76.36	77.73	76.99	78.03	84.52	81.85
Light	72.50	72.50	72.50	72.50	72.50	82.02	81.64
Oxidative	72.00	76.00	74.00	75.00	73.47	83.47	85.41
Salt	59.13	62.61	60.87	61.26	60.18	64.50	61.52
***K*‐mer + ACC**							
Cold	65.91	60.45	63.18	62.50	64.16	68.17	67.14
Drought	63.33	60.26	61.79	61.44	62.37	66.95	66.15
Heat	77.27	80.00	78.64	79.44	78.34	87.23	86.97
Light	67.50	73.33	70.42	71.68	69.53	77.76	78.15
Oxidative	75.20	63.20	69.20	67.14	70.94	77.83	77.87
Salt	60.29	64.06	62.17	62.65	61.45	64.95	62.18

*Note*. ACC, accuracy; auPRC, area under precision‐recall curve; auROC, area under receiver operating characteristics curve; K‐mer, K‐mer compositional features; Pre, precision; Sen, sensitivity; Spe, specificity.

### 3.4 Repeated cross‐validation analysis

**FIGURE 5:**
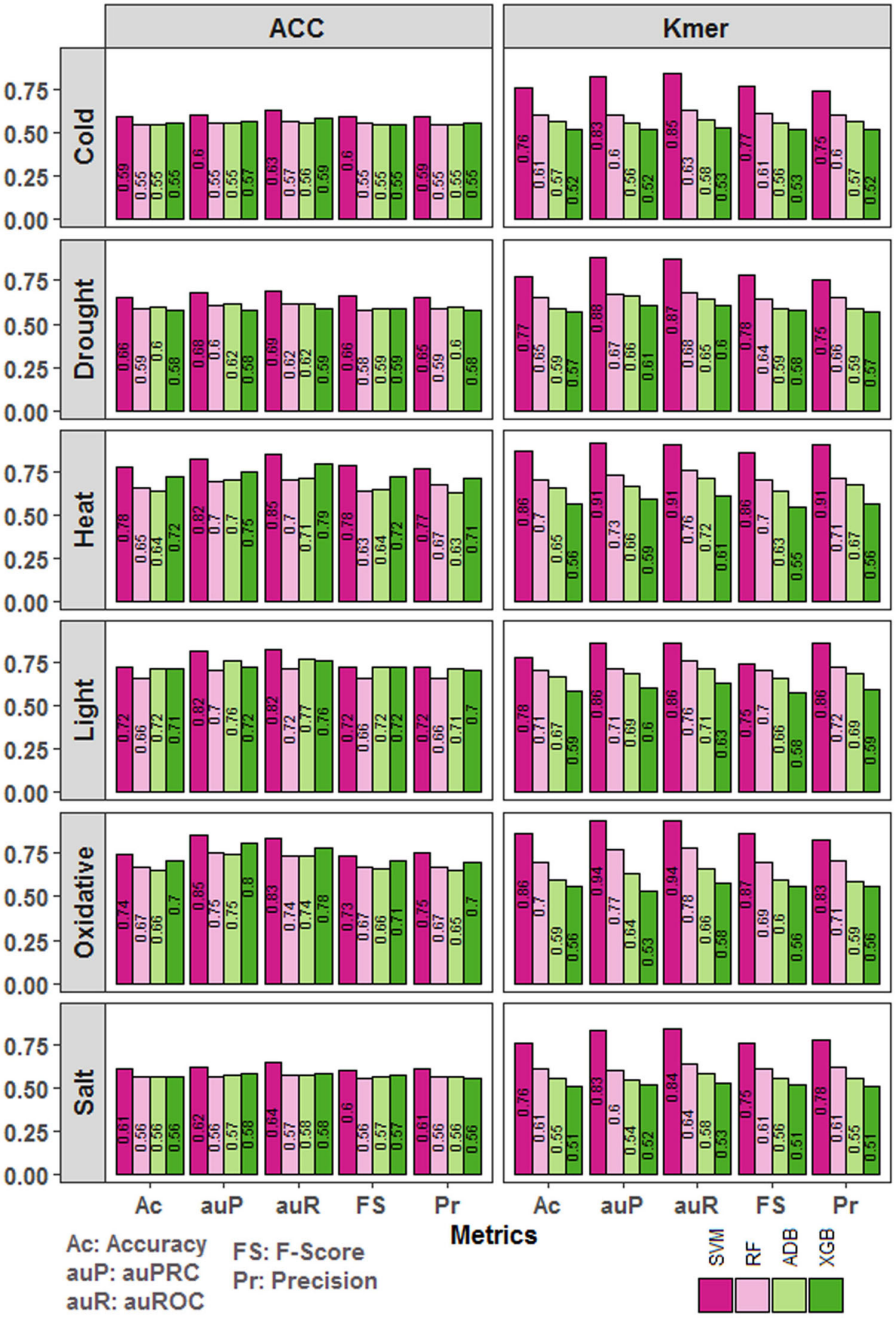
Comparison of support vector machine (SVM) performance metrics with the other machine‐learning methods. Performance metrics were computed following fivefold cross‐validation with the selected feature set

Earlier predictions, including feature selection using SVM‐RFE, were performed with a single balanced dataset comprising all the stress‐responsive sequences and the same number of non‐stress‐responsive sequences that were randomly taken from the available negative set. However, the negative set for each stress category was much larger than that of its positive counterpart (Table 2), and hence it may not be appropriate to adjudge the accuracy of the prediction model by taking only a subset of the negative set. Therefore, prediction analysis was further performed for each abiotic stress by following the repeated cross‐validation approach. In this approach, the experiment was repeated 100 times where for each experiment the same positive set, but a different negative set (randomly drawn) was used. Moreover, a fivefold cross‐validation approach was followed to measure the accuracy in each experiment, and the final accuracy was computed by taking the average over all the 100 experiments. Furthermore, SVM with *K*‐mer compositional features was only used for prediction purposes because of its higher accuracy than the other combinations of features and learning methods. Overall accuracy of 64.9, 65.6, 64.5, 61.3, 60.0, and 63.1% were obtained for cold, drought, heat, light, oxidative, and salt stresses, respectively, with *K*‐mer compositional features (Table 4). Also, the accuracy (∼76–86%) obtained with a single balanced dataset (Figure 5) were much higher than that obtained with a repeated cross‐validation approach (60–65%) (Table 4). In terms of auROC and auPRC, accuracy was >60% for *K*‐mer compositional features, except for oxidative stress (Table 4).

**TABLE 4:** Repeated cross‐validated performance metrics for support vector machine. The experiment was repeated 100 times, where the same positive set and a negative set containing an equal number of observations randomly drawn from the whole negative dataset were used. Each time the performance was measured following a fivefold cross‐validation technique, and the final metrics were computed by taking the average over all the 100 sets of the experiment

Stress	Sen^a^	Spe	Acc	Pre	*F* score	auROC	auPRC
Cold	0.633 ± 0.031	0.667 ± 0.035	0.649 ± 0.016	0.656 ± 0.020	0.644 ± 0.018	0.681 ± 0.018	0.649 ± 0.020
Drought	0.696 ± 0.019	0.616 ± 0.020	0.656 ± 0.016	0.644 ± 0.015	0.669 ± 0.015	0.699 ± 0.018	0.686 ± 0.019
Heat	0.661 ± 0.052	0.629 ± 0.073	0.645 ± 0.072	0.657 ± 0.099	0.644 ± 0.092	0.654 ± 0.108	0.669 ± 0.114
Light	0.567 ± 0.063	0.658 ± 0.071	0.613 ± 0.070	0.613 ± 0.097	0.583 ± 0.096	0.604 ± 0.096	0.607 ± 0.075
Oxidative	0.611 ± 0.061	0.589 ± 0.077	0.600 ± 0.085	0.581 ± 0.084	0.581 ± 0.093	0.588 ± 0.091	0.594 ± 0.076
Salt	0.611 ± 0.023	0.652 ± 0.029	0.631 ± 0.017	0.637 ± 0.020	0.623 ± 0.017	0.650 ± 0.023	0.617 ± 0.022

*Note*. ACC, accuracy; auPRC, area under precision‐recall curve ; auROC, area under receiver operating characteristics curve; Pre, precision; Sen, sensitivity; Spe, specificity.

### 3.5 Analysis with independent test set

It is clear from the analysis that SVM with *K*‐mer compositional features exhibited higher accuracy than the other features and ML algorithms. Thus, the SVM with *K*‐mer compositional features was employed to predict the test instances.

#### 3.5.1 Analysis with independent test Dataset I

The independent test set I comprises a total of 739 protein sequences belonging to two (613), three (116), and four (10) stress categories. The distribution of sequences over different stress combinations is provided in Supplemental Table S1. Out of 613 proteins responsive to two stresses, 364 (∼60%) sequences were correctly predicted, and among them, 126 sequences were correctly classified into both stress categories and the rest of the sequences into one of the two stress categories (Supplemental Figure S2). Similarly, for the three stresses category, 73 sequences (62.93%) were correctly predicted in at least one category (Supplemental Figure S2). Out of 10 instances corresponding to four stresses, eight sequences were correctly predicted into any of the four stresses (Supplemental Figure S2). The prediction accuracy was at par with that of the repeated cross‐validation accuracy.

#### 3.5.2 Analysis with independent test Dataset II

The independent test set II was prepared similar to how the positive and negative datasets were prepared. Further, we ensured that none of the test sequences were repeated in the training dataset. Additionally, the number of sequences for both positive and negative sets was kept almost the same. The negative set was prepared for each stress category by taking the same number of sequences from the rest of the five stress categories. For instance, the positive set for the cold stress contained 149 sequences belonging to cold stress alone, and the negative set was designed to contain 145 sequences from the rest of the five stress categories, including 29 sequences from each of them. The prediction accuracy for the independent test set II is given in Table 5. The overall accuracy for the cold, drought, heat, light, oxidative, and salt stress was 54.4, 54.9, 63.1, 58.2, 71.8, and 60.3%, respectively. It was also seen that the accuracies are similar to the repeated cross‐validation accuracies.

**TABLE 5:** Summary of the prediction accuracy for the independent dataset‐II. The support vector machine with selected *K*‐mer compositional features was used for the prediction of test instances. The accuracy was observed to be consistent with the repeated cross‐validation accuracy

Stress	No. positive	No. negative	Acc^a^	Pre	*F* score	auROC	auPRC
Cold	149	145	0.544	0.549	0.553	0.563	0.582
Drought	178	175	0.549	0.551	0.562	0.610	0.638
Heat	71	70	0.631	0.617	0.657	0.659	0.721
Light	83	80	0.582	0.586	0.601	0.638	0.691
Oxidative	142	140	0.718	0.656	0.769	0.855	0.858
Salt	213	210	0.603	0.613	0.592	0.613	0.603

*Note*. ACC, accuracy; auPRC, area under precision‐recall curve; auROC, area under receiver operating characteristics curve; Pre, precision. The ACC was computed as the average of the sensitivity and specificity.

### 3.6 Online prediction tool ASRpro

An online prediction server, ASRpro, was developed and made freely accessible (https://iasri‐sg.icar.gov.in/asrpro/) to allow the users, particularly those from noncomputational backgrounds, to use the prediction approach. The user interface has been designed with HTML, where the developed R code runs at the backend with the help of hypertext preprocessor upon submission of protein sequences in the FASTA format. The options of pasting and uploading the protein sequences in the FASTA file were provided. The prediction result for each sequence was displayed with the predicted stress category along with its probability. With the availability of the prediction server, the experimental biologists can easily get the candidates to test. The web server has been developed by following the guidelines of MIABi (minimum information about bioinformatics investigation) (Tan et al., 2010). The MIABi‐web server (MIABi‐WS) guidelines are provided in the Supplemental Material.

## 4 DISCUSSION

Abiotic stress is one of the major factors limiting crop productivity and threatening food security (Gürel et al., 2016; Zhu, 2016). Additionally, the geographical distributions of the plant species are also primarily affected by different environmental stresses. Since the environment is unpredictable, maintaining plant growth is challenging under different abiotic stresses (Pandey et al., 2017). Further, breeding crop cultivars with improved stress tolerance requires a better understanding of plant responses to different abiotic stresses. Moreover, discovering the key genes underlying plant responses to environmental stresses will play an important role in developing strategies for crop genetic improvement (Ma et al., 2012). Therefore, mining and identifying SRGs are important steps toward predicting the plant response under stress. Given the importance of identifying various SRGs, the present study offered a new computational tool for identifying the proteins associated with multiple abiotic stresses.

The generation of discriminatory features for the classification of biological sequences has a major role in achieving higher classification and prediction accuracy. This work generated ACC and *K*‐mer compositional features to map the protein sequences into numerical features that were subsequently used as input in ML algorithms for prediction. Predictions were made using three different feature sets: ACC, *K*‐mer, and ACC + *K*‐mer. The generated feature sets were larger, that is, 8,420 for *K*‐mer compositional features and 1,641 for ACC. Predictions with many irrelevant features may result in overprediction accuracy, and hence, a feature selection strategy was employed for selecting the important features. The SVM‐RFE technique was used here to select important features for predicting abiotic stress‐responsive proteins with higher accuracy. Further, a sample dataset comprising an equal number of instances from both positive and negative sequence classes was used for each stress category. The sizes of the selected feature sets were different for different stress categories for all three feature sets (ACC, *K*‐mer, and ACC + *K*‐mer). It was found that the sizes of the selected feature set were larger for the larger size dataset. For instance, the selected *K*‐mer compositional features were larger in number for cold, drought, and salt than for heat, light, and oxidative stresses. One of the probable reasons for this may be that a relatively larger feature set is required to achieve convergence in accuracy for the larger dataset. However, it is not always true. In other words, if the generated features are highly correlated, the resultant size of the selected feature set may be small.

Using the selected feature sets of *K*‐mer, ACC, and ACC + *K*‐mer, predictions were made with four different ML techniques: SVM, random forest, ADB, and XGB. The predictions were performed using the balanced dataset for each stress consisting of an equal number of instances from positive and negative datasets. The accuracy with *K*‐mer compositional features was found to be higher than that of ACC. However, the accuracy was not improved even after combining both the feature sets. Instead, the accuracy of ACC and ACC + *K*‐mer compositional features was at par despite the selected feature set of ACC + *K*‐mer being even larger than that of the *K*‐mer compositional feature set. Higher accuracy was observed with SVM than with the other three ML algorithms. One of the possible reasons may be that the features selected with SVM‐RFE may not be an optimal feature set for other ML techniques. It was also found that the accuracy was higher for the stresses with a smaller dataset (heat, light, and oxidative) than with the larger dataset (cold, drought, and salt). This may be because the cold, drought, and salt stress‐responsive sequences share a higher degree of similarity among themselves and a lesser degree of similarity with heat, light, and oxidative stress‐responsive sequences. The higher accuracy of *K*‐mer compositional features than the ACC features may be attributed to capturing a longer sequence order effect with the *K*‐mer compositional features. The presence of amino acid repeats would have a negative effect on prediction accuracy. However, a large number of *K*‐mer (8,420) compositional features have been used, and a change in the proportion of certain *K*‐mers will have less impact on prediction accuracy. Although, it is possible that a slight perturbation in *K*‐mer may turn a stress‐responsive protein predicted as a stress nonresponsive protein. At the same time, it is possible that changes in *K*‐mer may lead to a stress nonresponsive protein predicted as a stress‐responsive one. Nevertheless, including the dataset of more and more crop species would help to capture more variability (including repeats or duplication) and improve the accuracy of the model.

Since the size of the negative set was larger than that of the positive set for each stress category, a balanced dataset (with a randomly selected negative set of equal size to the positive set) was used for prediction for each stress. However, only using one subset of the negative set may not be sufficient to represent the whole negative set. In fact, it is not appropriate to adjudge the actual prediction accuracy of the learning algorithms with a single subset of the negative set. Thus, a repeated cross‐validation approach was adopted for measuring the prediction accuracy by repeating the experiments 100 times with each experiment performed with a balanced dataset comprising all the positive instances and a subset of the negative set with equal size randomly drawn from the available sequences. In other words, in each experiment, the same positive set and a different negative set were used. The repeated cross‐validation accuracy was lower than the accuracy obtained with a single balanced dataset. This may be because the features selected using a certain dataset may not be appropriate for other datasets as far as higher accuracy is concerned.

Since protein sequences were found to be associated with multiple stresses, a single predictor trained with multiple sequence classes is not meaningful. Although a multiclass predictor with seven different classes—six classes corresponding to six different stresses and one corresponding to the nonstress category—is possible, the prediction for the test instances can only be made to any of these classes in terms of the highest probability of prediction. Thus, instead of developing a single multiclass predictor, six different predictors were established for predicting the probabilities of each test instance belonging to six different stresses. The proposed learning model was further evaluated with an independent test set having sequences associated with two, three, and four abiotic stresses, where the accuracy was found to be consistent with the repeated cross‐validation accuracy.

The sequence datasets used in this study are mostly from *Arabidopsis thaliana* (L.) Heynh., although there are sequences from other plant species such as rice, wheat (*Triticum aestivum* L.), maize (*Zea mays* L.), barley (*Hordeum vulgare* L.), tomato (*Solanum lycopersicum* L.), cotton (*Gossypium hirsutum* L.), and many more. The present computational approach can be useful for the prediction of abiotic stress‐responsive proteins for other crops irrespective of variability in epigenetic changes. Nonetheless, being the first of its kind, the proposed approach is expected to supplement the existing efforts for the identification of SRGs.

The developed approach is freely available to the research community through an online prediction server ASRpro (https://iasri‐sg.icar.gov.in/asrpro/) that can be used to predict the stress type (with probability) that a given protein sequence may be associated with.

## AUTHOR CONTRIBUTIONS

Prabina Kumar Meher: Conceptualization; Data curation; Formal analysis; Funding acquisition; Investigation; Methodology; Project administration; Resources; Software; Supervision; Validation; Visualization; Writing‐original draft; Writing‐review & editing. Tanmaya Kumar Sahu: Data curation; Formal analysis; Resources; Software; Validation. Ajit Gupta: Formal analysis; Investigation; Project administration; Resources; Writing‐original draft; Writing‐review & editing. Anuj Kumar: Data curation; Formal analysis; Resources; Validation; Visualization. Sachin Rustgi: Conceptualization; Funding acquisition; Investigation; Project administration; Resources; Supervision; Writing‐original draft; Writing‐review & editing.

## CONFLICT OF INTEREST

The authors declare no conflict of interest.

## DATA AVAILABLITY STATEMENT

All the datasets used in this study have been collected from the public domain. The datasets are available online (https://iasri‐sg.icar.gov.in/asrpro/dataset.html).

## Supporting information

Supplemental Figure S1. Plots of auROC and auPRC with ACC and ACC+K‐mer features obtained with the SVM‐RFE feature selection approach.
Supplemental Figure S2. Chord diagrams show the number of observed and correctly predicted test instances of different stress categories.
Supplemental Table S1. Summary of the independent datasets.
