# Supplementary material for: ASRpro: A machine‐learning computational model for identifying proteins associated with multiple abiotic stress in plants
Source: Plant Genome. 2022 Sep 13;17(1):e20259. doi: 10.1002/tpg2.20259 (PMC12806878; doi:10.1002/tpg2.20259)
Supplement: Supplementary file 1 [file TPG2-17-e20259-s001.docx]

**Supplementary Figure S1.** Plots of auROC and auPRC with ACC and ACC+K-mer features obtained with the SVM-RFE feature selection approach.

**Supplementary Figure S2.** Chord diagrams show the number of observed and correctly predicted test instances of different stress categories. The prediction for the test instances was made by using the support vector machine trained with selected feature sets of respective stress categories.

**Supplementary Table S1.** Summary of the independent datasets.

| **Stress combinations** | #**Sequences** |
| --- | --- |
| Cold+Drought | 195 |
| Cold+Heat | 23 |
| Cold+Salt | 227 |
| Drought+Heat | 59 |
| Drought+Light | 23 |
| Heat+Salt | 44 |
| Light+Salt | 21 |
| Oxidative+Salt | 21 |
| Cold+Drought+Heat | 19 |
| Cold+Drought+Salt | 79 |
| Drought+Heat+Salt | 18 |
| Cold+Drought+Heat+Salt | 10 |
| Total number of sequences | 739 |

The MIABi guidelines for developing a web server (MIABi-WS)

**Scope –** MIABi-WS is the Minimum Information About a Bioinformatics Web Server which defines the minimum information to be reported for a new bioinformatics Web server.

**Purpose –** MIABi-WS contains information that can allow anyone suitably skilled in the art,

a) to reproduce the Web server with some effort (except in the case of a proprietary Web server - see below) and/or

b) to use the Web server, for the purposes of

- determining the veracity of the claims of the authors of such a Web server found in the scientific publication that cites its compliance, and
- verifying the validity the web server for the purposes of the scientific investigation citing its MIABi-compliant use, and
- reproducing the results which the publication that cites its compliance adduces.

**Protection of Intellectual Property –** Where a Web server is not intended for open source or open access, and that its content, though described as far as possible in compliance to MIABi, is classified proprietary and not revealed, then the MI disclosure shall include full reference to the source of these proprietary material and the mechanism for access or licensing or purchase, as the case may be, in order that the claims made by the publication can be verified or validated.

**Content –** MIABi-WS shall contain the following minimal information:

a) General descriptions

- Date stamp (as YYYY-MM-DD)
- Responsible person (or institutional role if more appropriate); provide name, affiliation and stable contact information
- Type of web server
- Description of its functionalities
- Platforms supported
- For all software and algorithms used in creating the Web server, the name, version and manufacturer
- Customizations made to that software and algorithm
- Availability of that software and algorithm
- Accessibility of the Web server, including mechanism of access (e.g. Internet)
- Data transformation and normalization technique
- Frequency of updates
- Overall research process, workflow pipelines and data flow diagrams where Appropriate

b) Input data

- File type and format

c) Input parameters

- All parameters used to create the new Web server to derive the claims made by the final results

d) Output of the process

- Types of search queries supported by the Web server, where applicable
- Process of the query procedure
- Output data types and formats
- Sequence (for identified nucleotides, peptides, or proteins)
- Quantitative scores (for identified nucleotides, peptides, proteins, pathways, etc)

e) Regression testing, performance and scalability

- Description of the training and test datasets to check the validity of the claims made by the Web server
- Description of the regression test process to verify the claims made by the Web server

f) Provenance

- All metadata regarding the provenance of the web server and where they are accessible for independent enquiry, audit, review, assessment, verification and validation.
